# Efficacy and Safety of Direct-Acting Antiviral Therapy in Patients With Chronic Hepatitis C Virus Infection: A Real-World Single-Center Experience in Tianjin, China

**DOI:** 10.3389/fphar.2020.00710

**Published:** 2020-05-19

**Authors:** Huan Xia, Chengzhen Lu, Yin Wang, Silvere D. Zaongo, Yue Hu, Yue Wu, Zhongfang Yan, Ping Ma

**Affiliations:** ^1^Department of Infectious Disease, Tianjin Second People’s Hospital, Tianjin, China; ^2^Department of Hepatology, Tianjin Second People’s Hospital, Tianjin, China; ^3^Department of Infectious Disease, Public Health Clinical Center of Chengdu, Chengdu, China; ^4^International School of Medicine, Tianjin Medical University, Tianjin, China; ^5^Tianjin Second People’s Hospital, Tianjin, China

**Keywords:** direct acting antivirals, hepatitis C, real-world experience, DAAs, HCV, China

## Abstract

**Objective:**

Toward the limited real-world data concerning the treatment response to brand direct-acting antiviral agents (DAAs) therapy, we proposed to evaluate the efficacy and safety of DAAs for the treatment of chronic hepatitis C virus (HCV) in mainland China.

**Methods:**

In this retrospective, single-center, cohort study, all HCV-infected adult patients treated with brand DAA drugs covered by Tianjin local health insurance (Apr 2018–Sept 2019) and responding to other specific inclusion criteria were recruited. The five available DAA regimens included sofosbuvir + ribavirin (SOF + RBV), elbasvir/grazoprevir (EBR/GZR), ombitasvir/paritaprevir/ritonavir/dasabuvir (OBV/PTV/r/DSV) ± RBV, daclatasvir + asunaprevir (DCV + ASV), and SOF + DCV ± RBV. Demographic, virologic, clinical, and adverse effects data obtained during and after DAAs treatment were collected. We evaluated the rate of sustained virological response at 12 weeks post-treatment (SVR12), the incidence of adverse effects, and assessed the factors associated with SVR12.

**Results:**

Four hundred ninety-four patients finished the treatment and completed the 12-week post-treatment follow-up. The overall SVR12 rate was estimated at 96.96%. SVR rates greater than 95% were achieved in most of the HCV genotypes with the exception of GT1a (0%), GT3a (93.33%), and GT3b (88.24%). SVR12 for patients treated with DCV + ASV, EBR/GZR, OBV/PTV/r/DSV ± RBV, SOF + DCV ± RBV, and SOF + RBV for 12 or 24 weeks was 86.67%, 100%, 98.11%, 97.56%, and 95.06%, respectively. Subjects with compensated cirrhosis (92.73%) and prior treatment experience (77.78%) had significantly lower SVR rates when compared to chronic hepatitis C (98.15%) and treatment-naive (97.69%) groups. In Tianjin, the available DAA regimens were generally well-tolerated, and not a single serious adverse event was reported.

**Conclusion:**

In this large real-life single-center HCV cohort from China, oral DAAs were highly effective and well-tolerated. Further and larger-scale studies are needed to evaluate their clinical safety and efficacy.

## Introduction

Globally, chronic hepatitis C virus (HCV) infection is a significant challenge to public health. In 2015, an estimated 71 million people were infected with HCV worldwide, and there were approximately 9.8 million HCV viremic people in China (Polaris Observatory, 2017). There, the infection is showing a significant increase in some provinces (Liu et al., 2018). In fact, according to the latest report from Tianjin Centers for Disease Control and Prevention, the incidence of chronic HCV in Tianjin was 5.87/100,000 in 2018, which was as much as 1.4-fold higher compared to the numbers estimated in 2016. In China, HCV is much more prevalent among older people (Liu et al., 2018), who are more likely to experience chronic liver disease. Long-term HCV infection is a leading cause of hepatic inflammation, extensive fibrosis, cirrhosis, hepatocellular carcinoma (HCC), and liver-related death (Polaris Observatory, 2017). The HCV-related diseases represent an immense health and economic burden in China.

The introduction of direct-acting antiviral agents (DAAs), with their high rates of sustained virological response (SVR) (European Association for the Study of the Liver, 2018), has revolutionized the management of chronic HCV infection. Thanks to DAAs, HCV can now be cured in most patients, even in those with advanced cirrhosis (Feld et al., 2015; Forns et al., 2017), genotype (GT) 3 (Kwo et al., 2017), and history of prior treatment failures (Feld et al., 2015; Lawitz et al., 2017). But the application of brand DAA drugs is limited in most regions of mainland China due to their expensive cost, different treatment guidelines, and reimbursement policies established by local governments (Bian et al., 2017). As a result, generic HCV drugs hold a high leading position in China. Fortunately, since April 2018, Tianjin local health insurance can cover HCV treatment (Bureau, 2018). Furthermore the brand DAAs used for the treatment include sofosbuvir (SOF), elbasvir/grazoprevir (EBR/GZR), ombitasvir, paritaprevir, ritonavir, dasabuvir (OBV/PTV/r/DSV), daclatasvir (DCV), and asunaprevir (ASV). Soon, the recently licensed SOF/velpatasvir (SOF/VEL) will be added to the reimbursement drug list. Tianjin health insurance reimburses up to $5,660 per HCV patient, which accounts for 85–90% of the cost (Bureau, 2018). The favorable reimbursement policy and early access to DAAs in Tianjin constitute a perfect condition to firstly report real-world experience with available brand DAAs in the treatment of Chinese HCV-infected patients.

Thus far, the results of real-world investigations on DAAs efficacy are mostly reported in western countries. In general, they present similar efficacy as observed in clinical trials (Saxena et al., 2017; Berg et al., 2019; Mera et al., 2019). These results also revealed the effectiveness of DAAs in some specific categories of patients (Saxena et al., 2017; Mera et al., 2019). Few real-life data have been reported in Asian countries except in Japan and Korea. Therefore, the purpose of this study was to assess the efficacy and safety of available brand DAAs in a sizeable real-life HCV patients cohort in Tianjin, China.

## Material and Methods

### Study Population and Antiviral Regimens

In this single-center—Tianjin Second People’s Hospital—retrospective real-world cohort study, patients meeting the following inclusion criteria were enrolled: (1) ≥18 years old; (2) a history of chronic HCV infection; (3) HCV GT 1, 2, 3, 6, unknown, or mixed; (4) with or without cirrhosis (compensated and decompensated); (5) treatment-naïve or treatment-experienced with interferon-based regimens; (6) negative results for antinuclear, anti-mitochondria, anti-smooth muscle autoantibodies; (7) with Tianjin local Medical Insurance; (8) treated with available brand DAAs covered by Tianjin local health insurance. Exclusion criteria were as listed: incomplete data, discontinued treatment, or loss during the 12-week post treatment follow-up. Ethical approval was obtained from the human medical ethics committee of Tianjin Second People’s Hospital and carried out following the principles of the Helsinki Declaration. Written informed consent was provided by each recruited patient.

At the treatment initiation, DAA containing regimens were chosen based on the current Asian-Pacific Association for the Study of the Liver (APASL) guidelines (Omata et al., 2016). During the study period, the available DAAs approved by Chinese government and covered by Tianjin local medical insurance were: (1) SOF (400 mg once daily) + ribavirin (RBV) daily (1,000 mg or 1,200 mg daily divided into three doses in patients who weighed < 75 kg or > 75 kg, respectively) for 12 or 24 weeks, (2) SOF (400 mg) + DCV (60 mg) ± RBV daily for 12 or 24 weeks, (3) EBR (50 mg)/GZR (100 mg) daily for 12 weeks, (4) OBV/PTV/r (25 mg/150 mg/100 mg once daily) + DSV (500 mg daily, divided into two doses) ± RBV for 12 weeks, and (5) DCV (60 mg) + ASV (100 mg) twice daily for 24 weeks. The use of RBV was determined by physicians depending on the practice guidelines and clinical indications for real-world settings. Patients were treated with different DAA regimens according to their clinical conditions.

### Data Collection

Demographic and baseline clinical characteristics, history of previous HCV treatment, and laboratory values were collected from the electronic medical records. The following clinical tests were completed at initial visits: anti-HCV, HCV RNA, HCV GT, serum hepatitis B virus (HBV) surface antigen, anti-human immunodeficiency virus (HIV), auto-antibodies, liver function, renal function, prothrombin time activity percentage, alpha-fetoprotein (AFP), thyroid function, chest-X ray, electrocardiography, abdominal imaging examinations, and tests of liver stiffness. Laboratory values were collected at baseline, week 12 and 24 [end of treatment (EOT)], then at week 12 after EOT.

Serum HCV RNA was tested using a Roche COBAS^®^ AmpliPrep/COBAS^®^ TaqMan^®^ HCV Quantitative Test (Roche Molecular Systems, Branchburg, NJ, USA; version 2.0). Anti-HCV reactivity was examined with a enzyme linked immunosorbent assay (Kehua Biotech, Shanghai, China). The HCV GT was sequenced and identified by a gene-sequencing assay.

Cirrhosis was defined by liver biopsy, whenever available, or based on clinical, laboratory, endoscopic, and radiological findings (i.e., abdominal ultrasound, computed tomography, magnetic resonance imaging, Fibroscan^®^). Decompensated cirrhosis was defined as the presence or history of variceal bleed, ascites, or hepatic encephalopathy. HCC was screened by at least two imaging tools, or by one imaging diagnostic modality plus a serum AFP level of at least 400 ng/ml.

### Evaluations of Efficacy and Safety

The primary efficacy endpoint was sustained virologic response (SVR12, which was defined as an undetectable HCV RNA viral loads < 15 IU/ml at week 12 after EOT). Adverse events (AEs) and serious adverse events that occurred both during and after treatment were recorded by physicians or nurses in charge. AEs related to DAAs therapy was defined as any unintended and unfavorable sign (including abnormal lab finding), symptom, or disease temporally associated with the use of DAA drugs. DAA drugs and these adverse reactions followed a chronological order (after initiation of DAAs treatment). All AEs were classified according to the Common Terminology Criteria for Adverse Events (CTCAE) Version 5.0 developed by the US National Cancer Institute (US NCI) (Health, N.I.o, 2017). Based on the tool, the severity of adverse event was classified according to unique clinical descriptions for each event.

### Statistical Analysis

Categorical variables were reported as frequencies (percentages); continuous variables were presented as median (interquartile range) or mean (standard deviation) as appropriate. Differences in categorical variables were assessed using the chi-square test or Fisher’s exact test. Continuous variables were compared using t-tests or Mann-Whitney *U* test. In the univariate analysis, chi-square and Fisher’s test were used for categorical variables when appropriate, and the odds ratio with 95% confidence intervals were calculated for SVR assessment. A two-tailed *P* value < 0.05 was considered statistically significant. All analyses were performed using SAS version 9.4 software (SAS Institute, Cary, NC, USA).

## Results

Between April 2018 and September 2019, 694 registered HCV infected Medicare patients were screened. After excluding those without treatment (n = 32), incomplete data (n = 42), discontinued treatment (n = 1), lost to follow up (n = 5), and patients with no post-treatment 12-week follow-up (n = 120): we recruited 494 patients who completed both DAAs treatment and 12-week follow-up compliance.

### Characteristics of the Study Population

The demographic and clinical characteristics of the patients were stratified according to SVR12 achievement status. The details are presented in **Table 1**. Overall, the mean age was 53.5 (± 13.07) years, 47.98% (237/494) were male, and 3.64% (18/494) were treated previously. Of the treatment-experienced patients, 72.22% (13/18) had previously received interferon-based regimen (**Table 1**).

**Table 1:** Demographic and clinical features based on SVR12 status.

	Total (n = 494)	No SVR12 (n = 15)	SVR12 (n = 479)	*p* value
Age in year, mean (SD)	53.50 (13.07)	55.47 (15.40)	53.44 (13.00)	0.2947
Male, n (%)	237 (47.98)	8 (53.33)	229 (47.81)	0.6732
Prior treatment experienced				
Overall, n (%)	18 (3.64)	4 (26.67)	14 (2.92)	<0.0001
Prior IFN-based regimen, n (%)	13 (2.63)	2 (13.33)	11 (2.30)	0.0554
Cirrhosis, n (%)				0.0264
Compensated	110 (22.26)	8 (53.33)	102 (21.34)	
Decompensated	6 (1.21)	0 (0.00)	6 (1.26)	
History of HCC, n (%)	10 (2.02)	1 (6.67)	9 (1.88)	0.2679
HIV/HCV or HBV/HCV co-infection, n (%)	20 (4.05)	2 (13.33)	18 (3.76)	0.235
Solid organ transplant recipients, n (%)	10 (2.02)	0 (0.00)	10 (2.09)	>0.9999
GT, n (%)				0.2737
GT1	355 (71.86)	9 (60.00)	346 (72.23)	
GT2	91 (18.42)	3 (20.00)	88 (18.37)	
GT3	32 (6.46)	3 (20.00)	29 (6.05)	
GT6	7 (1.42)	0 (0.00)	7 (1.46)	
unknown or mixed	9 (1.82)	0 (0.00)	9 (1.88)	
HCV RNA (Log_10_ IU/ml), mean (SD)	5.99 (0.96)	6.20 (0.75)	5.99 (0.97)	0.314
Liver stiffness measurement (LSM) (kPa), median (IQR)	9.00 (9.00)	13.5 (18)	6 (8)	0.3118
ALT (U/L), median (IQR)	43 (44)	41.5 (26)	43 (45)	0.9603
Bilirubin (*μ*mol/L), median (IQR)	14 (8)	14.5 (6)	14 (8)	0.3903
Albumin (g/L), mean (SD)	43.40 (6.12)	42.50 (5.54)	43.43 (6.14)	0.7132
AFP (ng/ml), median (IQR)	5 (6)	7 (20)	5 (6)	0.3723
eGFR (ml/min/1.73 m^2^), mean (SD)	97.17 (24.64)	104.00 (20.45)	97.00 (24.73)	0.588
Platelets (10^9^/L), median (IQR)	159 (101)	138 (109)	159 (100)	0.4332
Hemoglobin (g/L), mean (SD)	136.79 (24.14)	139.4 (24.98)	136.7 (24.14)	0.7661

Data expressed as mean (standard deviation) or median (Q3–Q1) or sample size and proportion (%). SD, standard deviation; IQR, interquartile range; SVR, sustained virologic response; IFN, interferon; HCC, hepatocellular carcinoma; HIV, human immunodeficiency virus; GT, genotype; HCV, hepatitis C virus; HBV, hepatitis B virus; ALT, alanine aminotransferase; AFP, alpha-fetoprotein; eGFR, estimated glomerular filtration rate.

**Figure 1:**
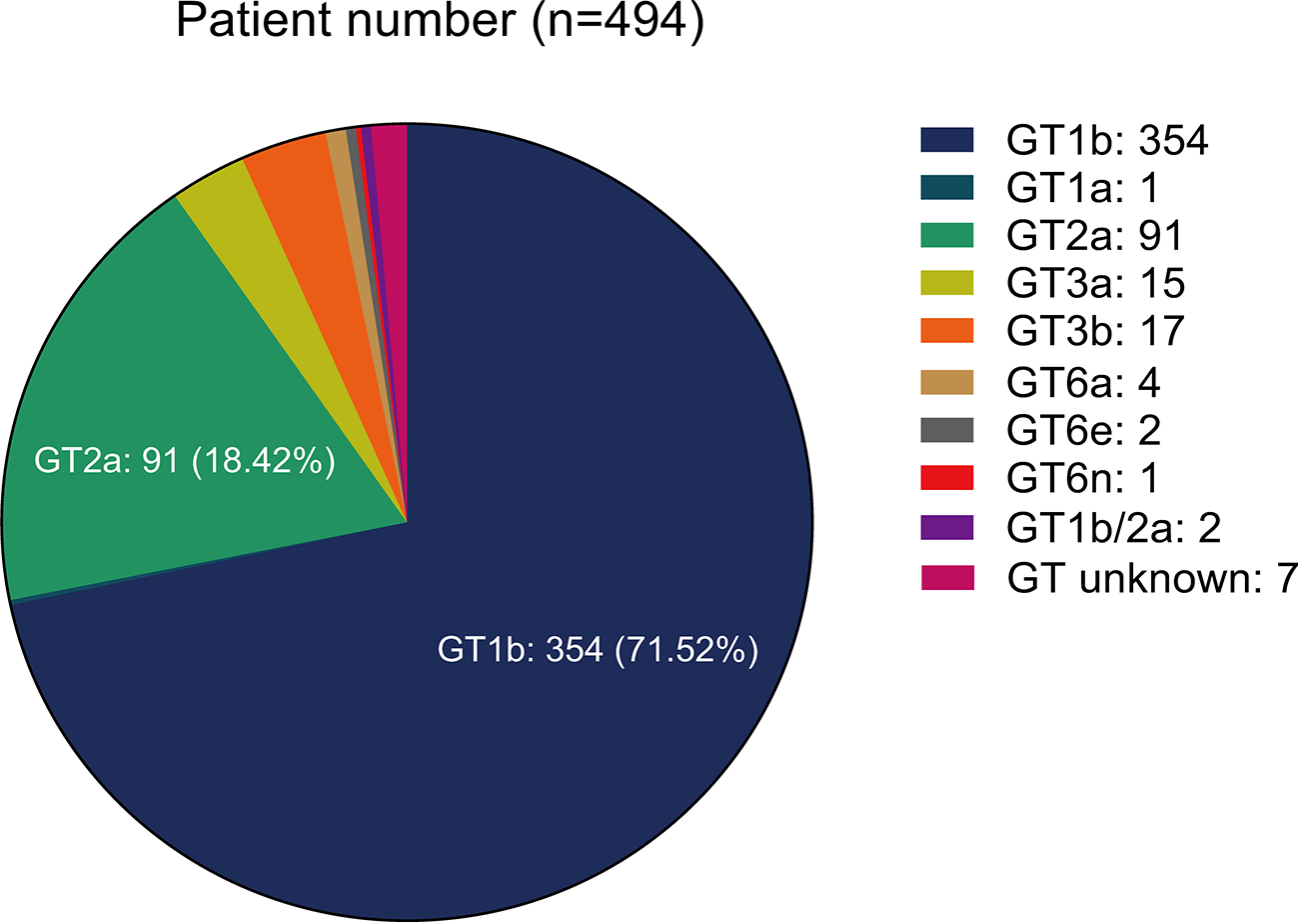
Hepatitis C virus (HCV) genotypes distribution.

From HCV GTs analysis, we noted the following distribution: 71.86% (355/494) of GT1(with 1 of 1a and 354 of 1b), 18.42% (91/494) of GT2 (all were of 2a), 6.46% (32/494) of GT3 (with 15 of 3a and 17 of 3b), 1.42% (7/494) of GT6 (with 4 of 6a, 2 of 6e, and 1 of 6n), and 1.82% (9/494) of unknown or mixed GT (**Figure 1**). More than 20% of the patients had cirrhosis (23.47%, 116/494). Among them, 5.17% (6/116) had a history of decompensated cirrhosis. Ten patients (10) out of 494 had a previous history of HCC (2.02%) while the double of this estimation represented the patients co-infected with HBV or HIV (4.05%, 20/494).

At baseline, median liver stiffness measurement (LSM) was 9 kPa. Besides, mean HCV-RNA and mean estimated glomerular filtration rate (eGFR) according to the MDRD (Modification of Diet in Renal Disease) formula were 5.99 Log_10_ IU/ml and 97.17 ml/min/1.73 m^2^, respectively. Most of the patient characteristics were similar in SVR12 achieved or not groups (*p* > 0.05). A statistically significant difference was noted when comparing the presence of cirrhosis (*p* = 0.0264), and prior-treatment experience status (*p* < 0.0001) between the aforementioned groups (see **Table 1**).

The treatment regimens use based on the GT profiles are shown in **Table 2**. Patients with GT1 used either OBV/PTV/r/DSV ± RBV (59.72%), EBR/GZR (17.75%), SOF + RBV (14.37%), SOF + DCV ± RBV (3.94%), or DCV + ASV (4.23%). On the other hand, patients with GT2-6 were either treated with SOF + RBV or SOD/DCV ± RBV while those harboring unknown or mixed GTs only received SOF + RBV.

**Table 2:** DAA regimens administered to hepatitis C virus (HCV) positive patients according to their genotype.

Regimens	Total	GT1	GT2	GT3	GT6	GT unknown or mixed
SOF + RBV	162 (32.79)	41 (14.37)	76 (83.52)	22 (68.75)	5 (71.43)	9 (100)
SOF + DCV ± RBV	41 (8.30)	14 (3.94)	15 (16.48)	10 (31.25)	2 (28.57)	0 (0.00)
EBR/GZR	64 (12.96)	63 (17.75)	0 (0.00)	0 (0.00)	0 (0.00)	0 (0.00)
OBV/PTV/r/DSV ± RBV	212 (42.91)	212 (59.72)	0 (0.00)	0 (0.00)	0 (0.00)	0 (0.00)
DCV + ASV	15 (3.04)	15 (4.23)	0 (0.00)	0 (0.00)	0 (0.00)	0 (0.00)

Data expressed as sample size and proportion (%).

DAAs, direct-acting antiviral agents; HCV, hepatitis C virus; GT, genotype; SOF, sofosbuvir; RBV, ribavirin; DCV, daclatasvir; EBR/GZR, elbasvir/grazoprevir; OBV/PTV/r/DSV, ombitasvir/paritaprevir/ritonavir and dasabuvir; ASV, asunaprevir.

### Treatment Efficacy

**Figure 2:**
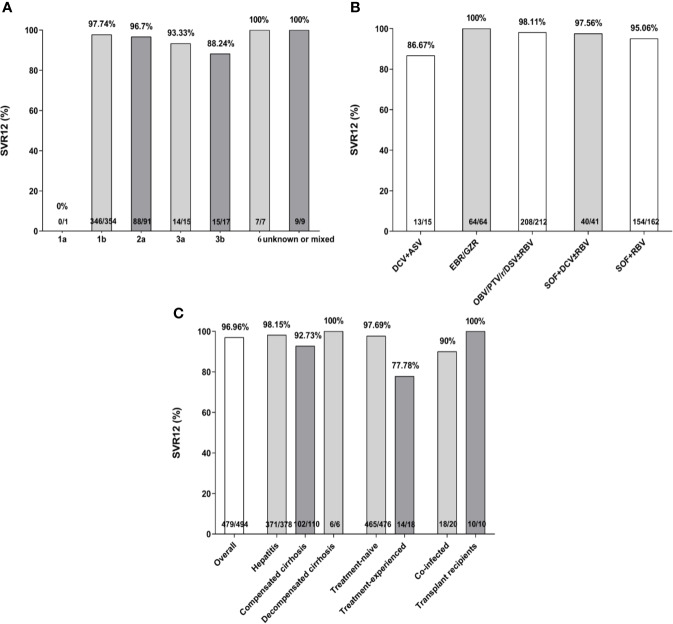
Sustained virological response (SVR) rates (%) according to hepatitis C virus (HCV) genotypes **(A)**, treatment regimens **(B)**, treatment history, liver stage, and specific sub-populations **(C)**. SVR, sustained virologic response; SOF, sofosbuvir; RBV, ribavirin; DCV, daclatasvir; EBR/GZR, elbasvir/grazoprevir; OBV/PTV/r/DSV, ombitasvir/paritaprevir/ritonavir and dasabuvir; ASV, asunaprevir.

There were 494 HCV infected patients with or without liver cirrhosis who completed the treatment. The overall SVR12 rate was estimated at 96.96% (479/494). Concerning HCV GTs, SVR rates greater than 95% were achieved in all GTs with the exception of GT1a (0%, 0/1), GT3a (93.33%, 14/15), and GT3b (88.24%, 15/17). The results revealed 86.67% (13/15), 100% (64/64), 98.11% (208/212), 97.56% (40/41), and 95.06% (154/162) of SVR12 in patients treated with DCV + ASV, EBR/GZR, OBV/PTV/r/DSV ± RBV, SOF + DCV ± RBV, and SOF + RBV for 12 or 24 weeks, respectively (**Figures 2A, B**). Subjects with compensated cirrhosis (92.73%, 102/110) and prior treatment experience (77.78%, 14/18) had relatively lower SVR rates when compared to chronic HCV (98.15%, 371/378) and treatment-naive (97.69%, 465/476) groups (**Figure 2C**). There were 90% and 100% of SVR rate for HBV or HIV co-infected patients (18/20) and transplant recipients (10/10), respectively.

### Factors Predicting Failure to Achieve SVR12

Overall, the absence of SVR12 was only associated with LSM (OR 1.043, 95%CI 1.006–1.082, *p* = 0.0221) and AFP (OR 1.002, 95%CI 1.000–1.003, *p* = 0.0303) in univariate analysis (**Table 3**). No differences in age, sex, HCV RNA, history of HCC, bilirubin, alanine aminotransferase (ALT), aspartate aminotransferase (AST), treatment regimens, and platelets were found between patients achieving or not SVR12. Besides, since GT 1b was the dominant GT, using a logistic regression, univariate and multivariate analyses were conducted to examine their potential influence on SVR12 onset. We concluded that GT1b was not able to predict the failure to achieve SVR12.

**Table 3:** Factors associated with SVR12.

Variables	Unadjusted		Adjusted	
	Univariate OR (95%CI)	*p* value	Multivariate OR (95%CI)	*p* value
Age (years)	1.012 (0.972,1.054)	0.5551	0.967 (0.912,1.024)	0.249
Male	1.248 (0.445,3.495)	0.6737	1.271 (0.276,5.852)	0.7586
HCV RNA (Log_10_ IU/ml)	1.296 (0.672,2.499)	0.4397	1.563 (0.627,3.897)	0.338
Liver stiffness measurement (LSM) (kPa)	1.043 (1.006,1.082)	0.0221	1.041 (0.990,1.093)	0.1156
History of HCC	3.723 (0.441,31.425)	0.2272	<0.001 (<0.001, >999.999)	0.9805
Bilirubin (*μ*mol/L)	1.000 (0.962,1.039)	0.9886	0.975 (0.869,1.094)	0.6676
ALT (U/L)	0.997 (0.985,1.009)	0.6293	0.975 (0.937,1.014)	0.1985
AST (U/L)	1.002 (0.989,1.015)	0.7618	1.006 (0.969,1.046)	0.7429
AFP (ng/ml)	1.002 (1.000,1.003)	0.0303	1.001 (0.999,1.004)	0.3683
Regimens				
SOF+RBV	Ref	Ref	Ref	Ref
SOF+DCV ± RBV	0.481 (0.058,3.961)	0.9615	0.950 (0.080,11.255)	0.9673
EBR/GZR	<0.001 (<0.001, >999.999)	0.9482	<0.001 (<0.001, >999.999)	0.9647
OBV/PTV/r/DSV ± RBV	0.370 (0.109,1.252)	0.9675	0.486 (0.089,2.654)	0.4046
ASV+DCV	2.962 (0.569,15.415)	0.9202	6.595 (0.724,60.036)	0.0941
Platelets (10^9^/L)	0.997 (0.989,1.004)	0.4019	0.997 (0.985,1.009)	0.6393

SVR, sustained virologic response; OR, odds ratio; CI, confidence intervals; HCV, hepatitis C virus; IFN, interferon; HCC, hepatocellular carcinoma; ALT, alanine aminotransferase; AST, aspartate aminotransferase; AFP, alpha-fetoprotein; SOF, sofosbuvir; RBV, ribavirin; DCV, daclatasvir; EBR/GZR, elbasvir/grazoprevir; OBV/PTV/r/DSV, ombitasvir/paritaprevir/ritonavir and dasabuvir; ASV, asunaprevir.

### Safety and Tolerability

Overall, adverse events were reported in 190 (38.5%) patients (**Table 4**). Fatigue (9.5%), anemia (7.5%), and dizziness (5.9%) were the most commonly encountered and were considered as drug-related in 71 (14.4%) participants. Adverse events ranged mostly between mild and/or moderate. Eleven (11, 2.2%) patients developed anemia due to RBV, which lead us to reduce its dosage. None of the patients developed severe adverse events (leading to discontinuation of the treatment) or died during the treatment (see **Table 4**). No HCC incidence was found throughout the study. Our observations indicated that DAA regimens were safe for HCV-infected patients who tolerated them well.

**Table 4:** Characteristics of the reported adverse events.

Adverse events	Total (n = 494)	SOF + RBV (n = 162)	SOF/DCV ± RBV (n = 41)	OBV/PTV/r/DSV ± RBV (n = 212)	EBR/GZR (n = 64)	DCV + ASV (n = 15)
Fatigue	47 (9.5)	15 (9.3)	8 (19.5)	21 (9.9)	2 (3.1)	1 (6.7)
Headache	14 (2.8)	4 (2.5)	2 (4.9)	8 (3.8)	0 (0)	0 (0)
Dizziness	29 (5.9)	7 (4.3)	3 (7.3)	13 (6.1)	5 (7.8)	1 (6.7)
Insomnia	14 (2.8)	5 (3.1)	4 (9.8)	2 (4.7)	2 (3.1)	1 (6.7)
Diarrhea	3 (0.6)	1 (0.6)	0 (0)	1 (0.5)	1 (1.6)	0 (0)
Nausea	11 (2.2)	2 (1.2)	1 (2.4)	5 (2.4)	2 (3.1)	1 (6.7)
Vomiting	11 (2.2)	0 (0)	1 (2.4)	7 (3.3)	3 (4.7)	0 (0)
Anemia	37 (7.5)	21 (12.9)	5 (12.2)	11 (5.2)	0 (0)	0 (0)
Abnormal liver function	24 (4.9)	8 (4.9)	3 (7.3)	10 (4.7)	1 (1.6)	2 (13.3)

Data expressed as sample size and proportion (%).

SOF, sofosbuvir; RBV, ribavirin; DCV, daclatasvir; EBR/GZR, elbasvir/grazoprevir; OBV/PTV/r/DSV, ombitasvir/paritaprevir/ritonavir and dasabuvir; ASV, asunaprevir.

## Discussion

In this retrospective single-center real-life study we assessed the outcome of HCV treatment with available brand DAAs from 2018 to 2019. Real-world data of HCV treatments are essential, especially to verify their efficacy and safety in daily practice outside the range of randomized controlled trials (Flisiak et al., 2017). Because of their high costs in China, brand DAAs are not easily accessible. Hence, few real-world data concerning their efficacy and safety were reported (Zeng et al., 2017; Hu et al., 2018). Moreover, many of these reports used generic DAA drugs, so the results may not represent the real outcomes. This could be an essential issue regarding HCV treatment, particularly in China which has the world’s largest HCV-infected population based on the estimated prevalence (Wei and Lok, 2014).

Overall SVR rates higher than 95% were observed across the majority of participants, except those having (1) compensated cirrhosis, (2) prior treatment experience, (3) GT1a and GT3 HCV infection, and (4) treatment with DCV + ASV. With the exception of SVR results from GT1a HCV-infected patients, our results were in accordance with previously published real-world data (Hong et al., 2018; Suzuki et al., 2018; Lobato et al., 2019). The lower than expected SVR rates in GT1a was probably due to the small sample size (only one patient). The patient had cirrhosis and was previously treated with EBR/GZR. Due to the distribution of HCV GTs in Asia (Ji et al., 2018) and China (Chen et al., 2017), HCV GT1a is relatively unusual. Therefore, our results were not sufficient for a meaningful estimation of SVR in HCV GT1a group.

Until the advent of the recently produced DAA (SOF/VEL, glecaprevir/pibrentasvir, SOF/VEL/voxilaprevir), genotype 3 was considered as difficult to cure (Nelson et al., 2015). In fact, many DAAs are less effective on this genotype in general and particularly on its subtype 3b (McPhee, 2019). Our data corroborate previous observations as genotype 3b patients had the lowest SVR12 rates if GT1a result is not considered. Most GT3 patients in our study were treated with SOF + RBV or SOF + DCV + RBV for 24 weeks. Our results are consistent with the findings of a phase 3 clinical trial in which SOF + RBV was administered during 24 weeks to treat Chinese patients with GT3 HCV infection (Huang et al., 2019). The investigators also noted that the presence of cirrhosis lowered SVR rates and the patient who relapsed had genotype 3b infection and cirrhosis. SVR12 rate was relatively low in patients receiving DCV + ASV, which was similar to previous real-world results (Hong et al., 2018; Ji et al., 2018; Itokawa et al., 2019). In our case, this observation was probably due to the very low power of the regimen characterized by its small sample size.

Moreover, the SVR12 rate of decompensated cirrhotic patients (6/6, 100%) was higher than compensated cirrhotic patients (102/110, 92.73%). This finding appears to be driven by either the small sample size, the extended 24-week regimen, or the addition of RBV. Notably, only about one-fifth of our cohort (22%) had cirrhosis, which was most likely due to the favorable reimbursement policy for DAAs therapy in Tianjin, China.

Our study, similarly to previous studies, demonstrated the efficacy of DAAs in the treatment of several sub-populations of HCV-infected individuals (HBV or HIV co-infections and solid organ transplant recipients) (Li et al., 2018; Zhang et al., 2019). Therefore, we suggest that HCV eradication with DAA regimens should not be withheld in these populations as all had excellent results.

Potential drug-drug interactions (DDIs) during DAA therapy should be considered especially when analyzing DAAs treatment response in patients with HIV or HCV co-infection or immunosuppression after solid organ transplantation. In our study, confirmation of DDIs before using DAAs was recommended. Subsequently, no clinically relevant DDIs was reported which implies that these five DAA regimens are safe even when they are co-administered with other drugs. However, more information are needed to confirm our statements and precautions should be taken.

This study has several limitations. Firstly, it was conducted in a restricted population. Therefore, information concerning the prevalence and distribution of viral and host factors that influence therapeutic outcomes are limited. Secondarily, DAA regimens only concerned brand agents covered by the Tianjin medical insurance programs. In regards to this, our findings cannot be generalized to other types of DAAs. Moreover, baseline NS5A/NS5B resistance-associated substitutions (RAS) testing was not performed. Having them tested would help to reduce a gap encountered in the literature. Actually, it is well-known that some of them, especially those related to NS5A region, were associated to lower response to therapy (Iio et al., 2017). However, some studies have reported that the prevalence of baseline RAS in Chinese HCV patients was relatively low and probably has not affected the SVR results (Wei et al., 2018a; Wei et al., 2018b). Besides, at the end of our data collection, there were a high number of patients (n = 120) who finished DAAs therapy but had not completed the 12-week post-treatment follow-up, their therapeutic effect (virological response) were not available and the current results should be further verified. The final limitation stands in the fact that, as the study describes real-world treatment outcome data, no control group of HCV-infected patients was included.

In summary, our study represents one of the largest cohorts of Chinese patients treated with various brand DAAs regimen available in a real-world setting. The overall SVR12 rates were comparable with that of international clinical trials, and the treatment was safe and well-tolerated. Liver stiffness measurement and AFP were predictors of not achieving SVR12. Future validation studies with a larger number of cases are required. Meanwhile, the current study could represent an important evidence leading to improvement of future strategies regarding the management and the use of DAAs in China.

## Data Availability Statement

The datasets analyzed in this article are not publicly available. The raw data required to support the findings of this study cannot be shared at this time as the data also forms part of an ongoing study. Requests to access the datasets should be directed to xiahuan1009@163.com.

## Ethics Statement

The study was conducted in accordance with the Declaration of Helsinki. Informed consent was obtained from all patients. The study was approved by the Human Medical Ethics Committee of Tianjin Second People’s Hospital.

## Author Contributions

HX and CL contributed equally as co-first authors. PM contributed to the design of this study. HX, YH, YWu, ZY, PM, and CL contributed to data collection. HX, YWa, SZ, and PM analyzed the data and wrote the article. All authors read and approved the final manuscript.

## Funding

This study was funded by the Key Project of Science and Technology Fund of Tianjin Health Commission (2014KR03 to PM), the 13th Five-year National Major Project for HIV/AIDS and Hepatitis B Control and Prevention, Chinese Ministry of Science and Technology (2017ZX10202102005004 to PM).

## Conflict of Interest

The authors declare that the research was conducted in the absence of any commercial or financial relationships that could be construed as a potential conflict of interest.
